# A New Perspective on the Health Benefits of Moderate Beer Consumption: Involvement of the Gut Microbiota

**DOI:** 10.3390/metabo9110272

**Published:** 2019-11-09

**Authors:** Mar Quesada-Molina, Araceli Muñoz-Garach, Francisco J. Tinahones, Isabel Moreno-Indias

**Affiliations:** 1 Department of Endocrinology and Nutrition, Virgen de la Victoria Hospital (IBIMA), Malaga University, 29010 Malaga, Spain; marquesadamolina@gmail.com (M.Q.-M.); aracelimugar@gmail.com (A.M.-G.); 2 Centro de Investigación Biomédica en Red de la Fisiopatología de la Obesidad y Nutrición (CIBEROBN), Instituto de Salud Carlos III (ISCIII), 29010 Málaga, Spain

**Keywords:** gut microbiota, beer, polyphenols, xanthohumol

## Abstract

Beer is the most widely consumed fermented beverage in the world. A moderate consumption of beer has been related to important healthy outcomes, although the mechanisms have not been fully understood. Beer contains only a few raw ingredients but transformations that occur during the brewing process turn beer into a beverage that is enriched in micronutrients. Beer also contains an important number of phenolic compounds and it could be considered to be a source of dietary polyphenols. On the other hand, gut microbiota is now attracting special attention due to its metabolic effects and as because polyphenols are known to interact with gut microbiota. Among others, ferulic acid, xanthohumol, catechins, epicatechins, proanthocyanidins, quercetin, and rutin are some of the beer polyphenols that have been related to microbiota. However, scarce literature exists about the effects of moderate beer consumption on gut microbiota. In this review, we focus on the relationship between beer polyphenols and gut microbiota, with special emphasis on the health outcomes.

## 1. Introduction

It has been estimated that approximately 2 billion people worldwide drink alcohol on a daily basis [1]. Although abuse of alcohol is unquestionably harmful, since the proposition of the “French paradox” in the early Nineties [2], possible positive effects of moderate consumption of alcoholic beverages on health have also been extensively investigated. The “French paradox” distinguished between fermented alcoholic beverages and spirits, in several studies the consumption of the former was found to have a positive effect on human health [3,4,5]. However, the debate remains open and a recent study claims that there is no minimum level of alcohol consumption that minimizes health problems [6].

Beneficial effects attributed to a moderate alcohol consumption should not be reduced to a unique single factor, like the amount of alcohol. In fact, most experimental trials have shown differences on health effects between spirits and fermented beverage when similar levels of alcohol consumption are compared [7,8,9], therefore, other additional factors must be considered. Fermented beverages like wine, cider, or beer contain significant amounts of healthy nutrients such as vitamins, polyphenols, or fiber. Wine, particularly red wine, is the fermented beverage that has received most attention due to its putative healthy properties. Moderate wine consumption has been related to a decrease in the risk of cardiovascular disease, type 2 diabetes (through an increase in insulin sensitivity), and favorable plasma lipid profiles [10,11,12]. These results are supported by both epidemiological studies and clinical trials.

However, beer is the most widely consumed fermented beverage in the world and has received less attention than wine. In 2006, nearly 1,700,000,000 hL of beer were drunk in the world [13]. Thus, beer consumption has been attracting increased interest. In this line, several clinical trials have reported that a moderate consumption of beer could mimic some of the previously reported health properties of red wine, among others, mainly due to the antioxidant [3] and anti-inflammatory effects of beer [14], as well as due to a reduced risk of cardiovascular diseases [3,14,15]. Similar to red wine, the mechanisms underlying these positive effects are not fully understood. Thus, in this review, we suggest a role as a mediator factor to the super-organs of gut microbiota.

## 2. Beer Is a Beverage Rich in Nutrients and Micronutrients

Beer is a fermented beverage produced with a scarce list of raw materials—water, malts (and its adjuncts), hops, and yeast, specifically *Saccharomyces cerevisiae*. Nevertheless, the technological steps of malting and brewing, and finally storage have important effects on the final composition of beer [16]. Malting is the first step of brewing and consists of mainly the cultivated barley (*Hordeum vulgare*), or other cereals, through controlled germination. The germinated malt is dried, grinded, and added to hot water in the mashing vessel, to produce a wort containing suitable amounts of fermentable sugars and yeast nutrients, as a result of enzymic hydrolysis or dissolving substances. This wort is boiled and mixed with hops (*Humulus lupulus*), a member of the *Cannabinaceae* family. In this step, α-acids humulones are transformed into trans-isohumulones, which provides the characteristic bitterness of beer. After this, a separation step is performed in order to remove the plant residues. At this moment, fermentation is initiated with the yeast strain and alcohol and carbon dioxide is produced. Finally, the maturation step during storage allows the formation of the particular aroma of each class of beer [17].

Thus, when the final product is ready, it contains hundreds of different compounds, some derived from raw materials that pass unchanged through brewing, while others are produced as a result of the technological process or during their maturation phase [18].

In beer, water represents more than 90% of its composition. Carbohydrates are the major nonvolatile component in beer with 3.3–4.4%, which comprise mainly dextrins (75–80%), monosaccharides, and oligosaccharides (20–30%), and pentosans (5–8%). Fermentation leads to the production of ethanol and a series of by-products, including other alcohols, carbonyl compounds, esters, aldehydes, and acids. The final alcohol content usually ranges from 1.0% to 6.0%, depending on the type of beer. The characteristic bitterness of a finished beer is derived from hops, especially from α-acids (humulones) and β-acids (lupulones). Iso-α-acids represent a varied concentration from 15 mg/L in typical American lagers to almost 100 mg/L in very bitter English ales [19]. In general, organic acids belong to yeast and bacterial fermentation; while the inorganic compounds found in beer are metal cations, trace metals, and anions, which influence the drink’s clarity and salty taste. The presence of these compounds is related to the initial raw materials, the brewing process or the packaging of the final product. Concentrations of inorganic components generally range from 0.5 to 2 g/L (for more information [16]).

Thus, beer is especially interesting because of its wide range of micronutrients. Beer includes a relatively significant content of folate (2.2–24.2 µg per bottle [20]) and choline (9.71 mg/100 mL [21]). Beer also contains trace amounts of minerals, such as calcium, iron, magnesium, phosphorus, potassium, sodium, zinc, copper, manganese, selenium, fluoride, and silicon [22].

Nevertheless, other components in beer are receiving increased attention because of their bioactive properties. This is the case of polyphenols, which confer special features to this beverage. Beer contains several polyphenols which are mainly derived from malt and hops [22].

These components have historically been associated with technological quality damage. However, their nutritional characteristics are being currently revised due to the interest in their potential health benefits [23].

## 3. Polyphenols and Health Benefits

Polyphenols are chemical and biologically active plant secondary metabolites derived from phenylalanine and tyrosine. This family of molecules is characterized by the presence of phenol (hydroxybenzene) units in their chemical structure. Plants synthesize these molecules as a response to several stress situations. These phytochemicals are ingested in food such as fruits, vegetables, and cereals. High levels of polyphenols are found in tea, coffee, wine, beer, or extra-virgin olive oil [24]. Some studies have attempted to link the observed health effects of a diet rich in fruits and vegetables with the content of polyphenols in these edible products [25].

Polyphenols display a wide range of biological activities that has been assessed in many studies, mainly in vitro studies with cell lines essays, but also with animal trials. Several advantageous effects have been reported, including decreases in markers of inflammation like IFM-γ, IL-1β, or NF-κB [26], and inhibitions of the expression of adhesion molecules, such as VCAM-1 and ICAM-1 [27]. Reduction of oxidative stress by polyphenols has also been demonstrated and has been related to a lower activity of cytochrome P450 [28] or through modification of gene expression via intracellular signaling cascades by, for example, reducing NF-κB or enhancing nuclear factor-like 2 (Nrf2) [29]. Furthermore, it has been reported that dietary phytochemicals improve anticancer markers [30], and might result in epigenetic modifications which confer protection against cancer [31]. Lastly, some trials have shown that some specific phenolic structures might have anti-diabetic [32] and antimicrobial properties [33]. Additionally, many articles have been written on polyphenols derived from hops and their properties during the last few years [34].

All these reported activities seem to be related and promoted by a synergistic effect of different polyphenols [10]. Particularly, polyphenols present in beer have been shown to exert a protective effect on the incidence of cardiovascular events, after moderate beer consumption [3]. The effects of alcohol and polyphenols from beer on atherosclerotic biomarkers have also been analyzed, and the results show that the phenolic content of beer reduces leukocyte adhesion molecules and inflammatory biomarkers [14]. Additionally, this polyphenolic content seems to elevate the number of circulating endothelial progenitor cells (EPC), a surrogate marker of vascular function and cumulative cardiovascular risk, after 4 weeks of beer consumption [7]. Conversely, alcohol mainly improves the lipid profile and reduces some plasma inflammatory biomarkers related to atherosclerosis [3,14].

Particularly, some specific beer-derived polyphenols exert interesting biological responses. They include anti-diabetic [8,35], anti-carcinogenic [36,37,38,39], and anti-inflammatory effects [40]. These specific polyphenols could be potential drugs used to treat related diseases. However, many of the mechanisms underlying these properties have not yet been elucidated. One hypothesis is that, at least partially, gut microbiota could mediate some of these beneficial effects. In this line, although not specially focused on beer polyphenols, several studies have been recently published emphasizing the role of gut microbiota for the bioavailability and physiological functions of dietary polyphenols [41,42].

## 4. Beer Is a Fermented Beverage with a Wide Range of Polyphenols

Absolute polyphenols content in beer is not higher than in other dietary products. According to the phenol explorer database, the total polyphenol content of beer ranges 12 to 52 mg/100 mL, which is in the same range as that of white wine (32 mg/100 mL), but is much lower than that in red wine (216 mg/100 mL) [43]. However, considering that the average beer intake is much higher than the consumption of red wine [10], relative contribution of beer to the daily polyphenol intake is probably the highest among fermented beverages.

Additionally, polyphenols refer to a wide family of biological compounds and its content level are only one of the variables to be considered. Even more important is the chemical diversity present in alcoholic beverages. Table 1 shows a classification of the polyphenols found in beer. Beer polyphenols are derived from malt (two-thirds) and hops (one-third) [18]. Polyphenols content depends on different factors, mainly crop variety [22] and environmental conditions of cultivation [25], as well as the technological brewing process followed [44].

Concerning bioactivity, beer polyphenols, especially those derived from hops, such as xanthohumol and isoxanthohumol, have a great potential [34]. The scientific literature has been mainly focused on the antioxidant activity of polyphenols for human health; however, polyphenols could also have other important properties that require more research.

## 5. Gut Microbiota and Polyphenols

The human intestine is inhabited by bacteria, archaea, fungi, protozoa, and viruses [46], which interact in a complex and dynamic symbiosis with the host. Around 10^14^ bacteria of more than 1000 different species has been identified in humans [47]. Gut microbiota survival is mainly maintained through the host diet, especially through the host-derived chemical compounds and metabolic byproducts excreted by the microbes themselves. Indeed, cooperation among microbial members, but also their competition for resources, are essential for the maintenance of the population within the gut environment [48]. However, the interactions of the gut microbiota with the host affect many aspects of human health and disease [49,50]. A complex network of different intestinal microbiota members is necessary for nutrient absorption in general and for the full polyphenols’ biotransformation, in particular [41].

Polyphenols are usually present and stored in plant tissues in the form of diverse derivatives, mostly as sugar O-conjugates [25], which confer extra structural stability [51]. Moreover, simple phenolics and their derivatives are usually covalently linked to polysaccharides in the plant cell wall [33]. Conjugation increases the chemical stability of polyphenols and allows high concentrations of these conjugated molecules in food and beverages. Nevertheless, their degree of structural complexity and polymerization will determine their absorption and bioavailability for the host [52,53]. Low molecular-weight polyphenols could be readily absorbed in the small intestine, while oligomeric and polymeric polyphenols will reach the colon with minimal chemical modifications [54]. Most dietary polyphenols belong to this last group and reach the colon. These complex polyphenols get in contact with the intestinal microbiota and are finally transformed. This conversion is often essential for absorption and bioavailability, modulating the biological activity of these dietary compounds [52,53]. These absorbed compounds enter the portal vein circulation towards final transformation in the liver, where other enzymatic activities are present. A complex network of different intestinal microbiota species is involved in this biochemical polyphenols processing [33]. To understand this gut microbiota–polyphenols interaction, it is essential to solve the mechanism of polyphenol bioactivity. It is a complex equation to solve because it is a two-way interaction and gut microbiota is also affected by polyphenolic compounds. These polyphenolic compounds and their metabolites, some produced by their own microbiota, are able to modulate the gut microbial community, mostly through the inhibition of pathogenic bacteria and the stimulation of beneficial bacteria [23,55]. Thus, gut microbiota bio-transforms polyphenols into their metabolites, resulting in a greater bioavailability of polyphenols, which, at once, are able to modulate the gut microbiota community mostly through the inhibition of pathogenic bacteria and the stimulation of beneficial members. In this last example, polyphenols might act as prebiotic metabolites which enrich the beneficial bacteria [23,55].

## 6. Interaction between Polyphenols Present in Fermented Alcoholic Beverages and Gut Microbiota

Toxicity of alcohol is involved in generating health complications. Conversely, alcohol is able to improve the lipid profile and reduce plasma inflammatory biomarkers related to atherosclerosis [3,14]. Alcohol might also produce an alteration of microbiota composition or function [47]. In a trial with chronic consumption of gin, an alcoholic beverage without any polyphenols, an increase in the abundances of *Clostridium* and *Clostridium hystoliticum* group was observed [9]. However, not all intake of alcoholic beverages yielded similar results. Other changes in the gut microbiota have been reported after consuming fermented or distilled beverages [9]. In polyphenol-rich alcoholic beverages such as red wine, chronic consumption resulted in an increased population of *Enterococcus*, *Prevotella*, *Bacteroides*, *Bifidobacterium*, *Bacteroides uniformis*, *Eggerthella lenta*, and *Blautia coccoides*–*Eubacterium rectale* groups [9]. Moreno-Indias et al. (2016) also observed a significant increase in the protectors of the gut mucosal barrier [5], such as *Bifidobacterium* spp. and *Lactobacillus* spp. This study was performed with regular red wine and de-alcoholized red wine intake periods by patients suffering from obese metabolic syndrome. *Bifidobacterium* spp. and *Lactobacillus* spp. are able to degrade phenolic compounds such as anthocyanin metabolites [56]. Additionally, *Lactobacillus* strains might advocate the normalization of the gut microbiota community and mitigate inflammation. Members of *Lactobacillus* are also able to reduce intestinal permeability, to improve the immunological functions of the intestine and to mitigate the intestinal inflammatory response as consequence [57]. On the other hand, *Bifidobacterium* has the capacity to produce lactate and acetate, as well as the ability to inhibit the growth of pathogenic bacteria [58]. Moreover, *Bifidobacterium* has been previously associated with the decrease of plasma cholesterol levels [59,60]. In this last study, independent to ethanol, wine polyphenols ingestion affected the growth of butyrate producers, namely the *Blautia coccoides–Eubacterium rectale* group, *Faecalibacterium prausnitzii*, and *Roseburia*. Additionally, this study showed that red wine polyphenols reduced the growth of the *Clostridium histolyticum* group, which includes important pathogens that are closely related to the progression of colon cancer and the onset of inflammatory bowel disease [61]. These results agree with those obtained by Tzounis et al., which make the essay with cocoa flavan-3-ols instead of wine [62].

## 7. Beer Polyphenols and Its Relationship with Gut Microbiota

As in other fermented beverages like wine, beer composition includes an important percentage of phenolic compounds. Table 2 summarizes some of the interactions between beer polyphenols and gut microbiota reported in the scientific literature. The most abundant hydroxycinnamic acid found in beer is ferulic acid, which mainly originates from barley [63]. However, many of the most interesting beer polyphenols belong to hops. Dried hops contain 4–14% polyphenols, mainly phenolic acids, prenylated chalcones, flavonoids, catechins, epicatechins, and proanthocyanidins [64]. Xanthohumol and its derivates mainly appear during brewing and particularly during the boiling of the wort. Isoxanthohumol, 8-prenylnaringenin, and 6- prenylnaringenin are the molecules most extensively studied and they have positively tested as displaying anti-obesity [65], anti-carcinogenic [66], and antidiabetic [67,68] activities. The bioactivity of these molecules is usually correlated with their amount, although in the case of xanthohumol derivatives, these metabolites exert a great activity at low concentrations [69,70,71].

The number of studies assessing the effects of moderate consumption of beer on the gut microbiota is low when compared with the number of clinical trials, epidemiological studies, and in vitro essays that show positive effects of this intake on human health. However, derivatives from hop polyphenols, such as isoxanthohumol, have been studied in relation to gut microbiota. In fact, isoxanthohumol is considered an accurate biomarker of beer consumption [86]. This molecule can be metabolized by gut microbiota to render 8-prenylnaringenin [72,73] through an O-demethylation that is carried out by *Eubacterium* species and particularly by *Eubacterium limosum* or *Eubacterium ramulus* that is responsible for this transformation [75]. The genus *Eubacterium* is one of the biggest butyric acid producers [87] and deserves further investigation. However, little attention has been paid to other hops compounds such as alpha- and beta-acids and their interaction with gut microbiota. Recently, Blatchford et al. (2019) used a pH-controlled anaerobic batch fermenter with a human fecal inoculum to investigate the interaction of a supercritical CO_2_ extract of hops on the human gut microbiota population [76]. Interestingly, they reported a decrease in butyrate concentrations aligned with a reduction of butyrate producers like *Eubacterium* and *Coprococcus*, as well as *Bifidobacterium*. Moreover, they found an increase of *Enterobacteriaceae* and *Akkermansia* [76].

Other studies have focused on ferulic acid, which is the most abundant polyphenol in beer with a concentration of 6.5 mg/L [10]. Six bacterial isolates from human feces have been reported to be able to release ferulic acid from its ethyl ester in vitro: *Escherichia coli* (three isolates), *Bifidobacterium lactis*, and *Lactobacillus gasseri* (two strains) [77]. On the other hand, rats in vivo essays showed that ferulic acid was related to an increase in bacterial richness and diversity [78,79]. This molecule is part of dietary fiber, the fundamental indigestible portion of food for the development of gut microbiota and especially for those members related to beneficial outcomes. Ferulic acid supplementation is able to modulate the ratio of Firmicutes to Bacteroidetes, as well as the production of indole-3-acetic acid in a nonalcoholic fatty liver disease model of mouse [80].

Some flavan-3-ols, such as catechins and epicatechins, are also found at high concentrations in beer (around 5.4 and 3.3 mg/L, respectively [10]). *Eggerthella lenta* and *Flavonifractor plautii* are able to biotransform dietary catechins into valerolactones and hydroxyvaleric acid metabolites [64]. Among the bioactive effects reported, it has been shown that epicatechin, catechin, 3-O-methylgallic acid, gallic acid, and caffeic acid are able to repress *Clostridium perfringens, Clostridium difficile*, and *Bacteroides* spp. growth, while other anaerobes commensal, such as *Clostridium* spp., *Bifidobacterium* spp. or *Lactobacillus* spp. were less negatively affected [55]. *Flavonifractor plautii* is also able to convert B type proanthocyanidins into valerolactones [88]. Conversely, proanthocyanidins extracts in humans, promoted the growth of *Akkermansia muciniphila* and improved diet-induced obesity, insulin resistance, and metabolic syndrome [89].

Finally, other flavonols present in beer are quercetin and rutin. *Butyrivibrio* spp. from ruminal fluid has the ability to cleave the C ring of rutin. In contrast, quercetin is cleaved by *Eubacterium oxidoreducens*, which was recovered from the bovine rumen [83]. Likewise, isolates of *Clostridium orbiscindens* strains from humans were found to be capable of cleaving the C ring of quercetin at the bond between the 3- and 4-positions [90].

## 8. Beer Polyphenols, Relationship with Gut Microbiota, and Health Benefits

The interaction among polyphenols present in beer and gut microbiota has recently been reported to have important health benefits, when changes in gut microbiota was analyzed after a moderate consumption of beer. Hernández-Quiroz et al. (2019) have recently reported a proliferation of Bacteroidetes with respect to Firmicutes, after a month of consuming an alcoholic or non-alcoholic beer on a daily basis [91]. These authors registered a higher diversity after the non-alcoholic beer, something that was translated into a decrease in fasting blood serum glucose and an increase in functional β cells. These results were not observed after the consumption of the alcoholic beer. Although it was not directly measured, it was concluded by the authors that health improvements were related to the biologically active polyphenol and phenolic acids [91].

Thus, to investigate the role of the polyphenols present in beer on health outcomes, through their gut–microbiota interactions, requires systemic approaches; meanwhile this action needs to be inferred. It has been reported that metabolites derived from hop have a positive effect on different health variables and they could also affect the gut microbiota populations. In this line, a study in mice found that tetra-hidro iso-alpha acids from hop, apart from reducing body weight gain, were able to reduce the fat mass, to alleviate glucose intolerance and to normalize insulin sensitivity markers in obese and diabetic mice [40]. These promising results were also related to gut microbiota, particularly to a reduction in plasma lipopolyshaccaride (LPS) levels and gut permeability, as well as higher intestinal tight junction proteins (Zonula occludens-1 and occludin), anti-inflammatory cytokine interleukin-10, and a decrease in pro-inflammatory cytokine granulocyte colony-stimulating factor [40]. Additionally, a recent study in DIO mice have revealed that chronic iso-alpha acid treatment altered enteroendocrine hormone levels and bile acid homeostasis and stimulated sustained GLP-1 release. As a result, this chronic treatment generated mass and weight loss, increased the energy expenditure, improved glucose tolerance and insulin sensitivity, and recovered the plasma lipids and inflammatory markers [67]. Many other studies have been developed with polyphenols from hops and their derivates. Two recent publications revealed that xanthohumol and 8-prenylnaringenin could ameliorate diabetic-associated metabolic disturbances by regulating glucose and lipid pathways [8,92]. On the other hand, several in vitro studies with human cancer cell lines have evidenced an anticancer activity, in both xanthohumol and 8-prenylnaringenin, with a decreased proliferation and invasion in colon [92], breast [38], prostate [93], and ovarian [38] cancers. Furthermore, a recent article has shown that the antiproliferative activity against human cancer cell lines of these molecules is very selective and dependent on the prenyl group, with respect to antiproliferative activity, as naringenin exerted a low activity [69]. However, beyond the fact that gut microbiota activates these phytoestrogens [72], no other relationships have been established between xanthohumol, gut microbiota, and cancer. It would be interesting to verify the relationship with the gut microbiota in order to decipher mechanisms subjacent to these actions.

Furthermore, other polyphenols present in beer has been related positively with health. The flavonol quercetin counteracted gut microbiota dysbiosis in rats fed with a high-fat sucrose diet and it also prevented body-weight gain, reduced serum insulin, attenuated the Firmicutes/Bacteroidetes ratio, and inhibited the growth of bacteria associated with diet-induced obesity [88]. Conversely, tannins like catechin, epicatechin, and proanthocyanidin have been reported to attenuate the overexpression of NF-κB, AMPK, TGF-β, PARP, and IL-6, factors which are involved in the progression of diabetic complications [94].

## 9. Conclusions

The available scientific literature reviewed in this manuscript supports that a complex interaction between polyphenols and gut microbiota could play a significant role on the healthy benefits that moderate beer consumption seems to provide. However, extensive experimental research is needed to confirm this hypothesis and to elucidate more details of this interaction. In fact, along this line, the recently available study by Hernández-Quiroz et al. (2019) has proposed this hypothesis to discuss their results [91]. Until now, beer is considered to be a popular refreshing beverage, but little attention has been given to the role that its bioactive components could exert on health, mainly the diverse polyphenols components. This review and other research reports showed that further investigations are needed, focusing on the causes of the health implications associated to xanthohumol and other derivatives from hop polyphenols, since the published results seems very promising. More research is also needed on the role of the more abundant polyphenols present in the beer, such as ferulic acid, to get a better knowledge on their interaction with gut microbiota.

## Figures and Tables

**Table 1:** Classification of the phenolic compounds in beer.

Class	Group	Congeners	Concentration
Monophenols	Phenolic alcohols	Tyrosol	3–40 mg/L
	Phenolic acids	p-coumaric acid, ferulic, vanillic, gallic, caffeic, syringic, sinapic acids, etc.	10–30 mg/L (including esters and glycosides)
	Phenolic amines and amino acids	Hordenine, tyramine, *N*-methyltyramine, tyrosamine, tyrosine	10–20 mg/L (3–8 mg/L as tyrosine)
Monomeric polyphenols	Flavonoids catechines (flavan-3-ol)	(+) catechin (+) epicatechin (possibly other isomers)	0.5–13 mg/L 1–10 mg/L
	Prenylated flavonoid	Xanthohumol, isoxanthohumol, etc.	0.002–3.5 mg/L
	α-acids and iso-α-acids	Cohumulone, n-humulone, adhumulone, and iso-cohumulone, iso-n-humulone, iso-adhumulone	2.3–100 mg/L
	Anthocyanogens	Leucocyanidin	4–80 mg/L
	Flavonols	Quercetin, kaempferol, myricetin (occur as glycosides), rutin, etc.	less than 10 mg/L
Condensed polyphenols		Dimeric catechins	5–8 mg/L
		Trimers	less than 1 mg/L

Adapted data from [45] and [18].

**Table 2:** Relationship between polyphenols within beer and gut microbiota.

Class	Phenolic Compound	Metabolite	Responsible Intestinal Microbiota and Implication	Experimental Conditions, Methodology and Tested Organism	Reference
Prenylated flavonoids	Isoxanthohumol	8-prenylnaringenin	*Eubacterium limosum*	In vitro (fecal human culture + isolate). HPLC Analysis *In vivo* (human and mouse) SHIME run, real-time PCR, HPLC analysis	Possemiers et al. 2005 [72] Possemiers et al. 2006 [73] Possemiers et al. 2008 [74]
	Xanthohumol	α,β-dihydroxanthohumol	*Eubacterium ramulus*	In vitro (bacterial culture of *E. limosum* and *E. ramulus*)	Paraiso et al. 2019 [75]
Bitter acids	α- and β-acids	Cohumulone, adhumulone, colupulone, lupulone, adlupulone	Increase of *Enterobacteriaceae* and *Akkermansia* Decrease of butyrate producers: *Eubacterium* and *Coprococcus* as well as *Bifidobacterium*	Hops extract fermentation in pH-controlled anaerobic batch fermenter with a human fecal inoculum 16SrRNA sequencing and qPCR	Blatchford et al. 2019 [76]
Monophenol. Phenolic acids	Ferulic acid		*Escherichia coli Bifidobacterium lactis* *Lactobacillus gasseri* Increase the α-diversity	In vitro (fecal human culture + isolate). 16SrRNA sequencing *In vivo* (mouse, dietary fiber from barley malts) *In vivo* (mouse). 16S rRNA sequencing	Couteau et al. 2001 [77] Teixeira et al. 2018 [78] Ou et al. 2016 [79]
		indole-3-acetic acid	Modulates ratio Firmicutes/Bacteroidetes	In vivo (mouse). 16S rRNA sequencing	Ma et al. 2019 [80]
Monomeric polyphenols. Flavan-3-ol	Catechin and Epicatechin	1-(3,4-dihydroxy-phenyl)-3-(2,4,6-trihydroxyphenyl)propan-2-ol 5-(3,4-Dihydroxyphenyl)-g-valerolactone 4-Hydroxy-5-(3,4-dihydroxy-phenyl)valeric acid	*Eggerthella lenta* *Flavonifractor plautiia*	In vitro (fecal human culture + isolate). 16SrRNA sequencing In vitro (fecal rats culture + isolate) 16SrRNA sequencing LC/MS and LC/MS/MS analyses	Kutschera et al. 2011 [81] Takagaki et al. 2015 [82]
Condensed polyphenols. Dimeric catechins	Proanthocyanidins	1-(3, 4, 5-trihydroxyphenyl)-3-(2, 4, 6-trihydroxyphenyl)propan-2-ol 1-(3, 5-dihy-droxyphenyl)-3-(2, 4, 6-trihydroxyphenyl)propan-2-ol	*Adlercreutzia equolifaciens* *Eggerthella lenta*	In vitro (fecal rats culture + isolate) 16SrRNA sequencing LC/MS and LC/MS/MS analyses	Takagaki et al. 2015 [82]
Monomeric polyphenols. Flavonols	Quercetin	3,4-dihydroxyphenylacetate	*Eubacterium oxidoreducens* Decrease: *Firmicutes/Bacteroidetes* Decrease: *Erysipelotrichaceae* *Bacillus* *Eubacterium cylindroides*	In vitro (Bacterial culture). Thin layer chromatography (TLC) *In vivo* (mouse). polymerase chain reaction (PCR) and bacterial 16SrDNA pyrosequencing	Krumholz & Bryant 1986 [83] Etxeberria et al. 2015 [84]
	Rutin	3,4-dihydroxy-benzaldehyde 3,4-dihydroxyphenylacetic acid	*Butyrivibrio*	In vitro (Bacterial culture) Chromatography (Sheep)	Krishnamurty et al. 1970 [85]
